# Association between medication complexity and follow-up care attendance: insights from a retrospective multicenter cohort study across 1,223 Chinese hospitals

**DOI:** 10.3389/fphar.2024.1448986

**Published:** 2024-07-29

**Authors:** Xuedi Ma, Yang Wang, Yongwu Chen, Yufei Lian, Xiaoyu Zhao, Xuan He, Yue Qiu, Sheng Han, Lihong Liu, Chen Wang

**Affiliations:** ^1^ School of Population Medicine and Public Health, Chinese Academy of Medical Sciences and Peking Union Medical College, Beijing, China; ^2^ State Key Laboratory of Cardiovascular Disease, Department of Medical Research and Biometrics Center, Fuwai Hospital, National Center for Cardiovascular Diseases, Chinese Academy of Medical Sciences and Peking Union Medical College, Beijing, China; ^3^ Department of Pharmacy, The First Affiliated Hospital of University of Science and Technology of China, Division of Life Sciences and Medicine, University of Science and Technology of China, Hefei, Anhui, China; ^4^ Department of Pharmacy, Hebei General Hospital, Shijiazhuang, Hebei, China; ^5^ Department of Pharmacy, The First Affiliated Hospital of Zhengzhou University, Zhengzhou, Henan, China; ^6^ Department of Pharmacy, Ruijin Hospital, Shanghai Jiao Tong University School of Medicine, Shanghai, China; ^7^ Institute for Hospital Management, Tsinghua University, Beijing, China; ^8^ International Research Center for Medicinal Administration, School of Pharmaceutical Sciences, Peking University, Beijing, China; ^9^ Department of Pharmacy, China-Japan Friendship Hospital, Beijing, China; ^10^ Department of Pulmonary and Critical Care Medicine, National Center for Respiratory Medicine, State Key Laboratory of Respiratory Health and Multimorbidity, National Clinical Research Center for Respiratory Diseases, Beijing, China; ^11^ Center of Respiratory Medicine, China-Japan Friendship Hospital, Institute of Respiratory Medicine, Chinese Academy of Medical Sciences, Beijing, China

**Keywords:** follow-up care attendance, medication regimen complexity, medication management, chronic disease management, chronic obstructive pulmonary disease

## Abstract

**Background:**

Patients with Chronic Obstructive Pulmonary Disease (COPD) frequently face substantial medication burdens. Follow-up care on medication management is critical in achieving disease control. This study aimed to analyze the complexity of COPD-specific medication and determine how it impacted patients’ attendance on follow-up care.

**Methods:**

This multicenter study includes patients with COPD from 1,223 hospitals across 29 provinces in China from January 2021 to November 2022. The medication Regimen Complexity Index (MRCI) score was used to measure COPD-specific medication complexity. The association between medication complexity and follow-up care attendance was evaluated using the Cox Proportional Hazard Model.

**Results:**

Among 16,684 patients, only 2,306 (13.8%) returned for follow-up medication management. 20.3% of the patients had high complex medication regimen (MRCI score >15.0). The analysis revealed that compared to those with less complex regimens, patients with more complex medication regimens were significantly less likely to attend the follow-up medication care, with a Hazard Ratio (HR) of 0.82 (95% Confidence Interval [CI], 0.74–0.91). Specifically, patients with more complex dosage forms were 51% less likely to attend the follow-up care (95% CI, 0.43–0.57). This pattern was especially marked among male patients, patients younger than 65 years, and those without comorbid conditions.

**Conclusion:**

Higher medication complexity was associated with a decreased likelihood of attending follow-up care. To promote care continuity in chronic disease management, individuals with complex medication regimens should be prioritized for enhanced education. Furthermore, pharmacists collaborating with respiratory physicians to deprescribe and simplify dosage forms should be considered in the disease management process.

## 1 Introduction

Follow-up care plays a critical role in chronic disease management by monitoring disease progression, offering patient education and support, and effectively managing medication (Jacobs et al., 2017; Silver et al., 2017; Venkatesan, 2024). Despite the recognized importance of care continuity in managing chronic diseases, high non-attendance rates persist (Silver et al., 2017; Kim et al., 2024; Wong et al., 2024). Such non-attendance can lead to lower medication adherence, worse health outcomes, and disease exacerbation over time (Andersen et al., 2000; Killaspy et al., 2000; Chen et al., 2013).

For the management of Chronic Obstructive Pulmonary Disease (COPD), the importance of follow-up care cannot be overstated. Patients with COPD frequently face the challenges associated with managing multiple medications and complex dose formulations (Franssen et al., 2011; Negewo et al., 2017). This complexity often results in poor medication adherence (Bogart et al., 2019; Jarab et al., 2019; Yu et al., 2020), incorrect medication usage (Chrystyn et al., 2017; Ruihuan et al., 2023), suboptimal disease management (Alves-Conceição et al., 2020), and adverse health outcomes (Varghese et al., 2024). Follow-up visits present an effective solution to these challenges, as providing an evaluation of medication efficacy and safety while also monitoring medication usage and adherence. Consequently, the significance of follow-up care is consistently highlighted within COPD management guidelines (Sandelowsky et al., 2022; Venkatesan, 2024). Research further underscored the value of follow-up care, revealing that patients who engaged in follow-up care for COPD experienced fewer all-cause readmissions, emergency department visits, and instances of mortality (Health Quality Ontario, 2017). Notably, the positive impacts of follow-up care were especially significant in aspects related to medication management (Fromer, 2011; Luder et al., 2015; Saxena et al., 2022). Despite these advantages, the rate of follow-up care attendance among patients with COPD remained low, ranging between 30% and 40% (Gavish et al., 2015; Sandelowsky et al., 2022; Saxena et al., 2022).

An important question arising from this context is how to enhance patient attendance for follow-up care. While providing reminders has been an intuitive and effective strategy (Sawyer et al., 2002), several published studies have delved into various factors that impacted follow-up care attendance. These factors span a wide range, including patient characteristics such as age, gender and comorbid conditions (Sharma et al., 2010; Kim et al., 2024), aspects of service delivery like providers’ education and communication skills (Jacobs et al., 2017), and healthcare resources coverage, including insurance coverage (Anderson et al., 2024) and healthcare access (Sharma et al., 2010; Gavish et al., 2015; Jacobs et al., 2017). Nonetheless, most of this research has been confined to post-discharge follow-up visits during COPD management, leaving the examination of outpatient follow-up care attendance less explored. Another vital yet under investigated dimension is the influence of medication complexity on follow-up care attendance. Patients with chronic diseases often face substantial medication burdens to control the primary disease and comorbidities (Molist-Brunet et al., 2022). Medication complexity varies significantly among patients, while in many cases, the simplification of medication regimens is feasible. A deeper understanding of how the complexity of medication regimens influences follow-up care attendance in outpatient setting could be instrumental in enhancing disease management optimally.

This study aimed to evaluate the impact of medication complexity on follow-up care attendance within the Cough and Wheeze Pharmaceutical Care Clinic (CWPC). These clinics were designed to offer comprehensive and continuous medication management. We focused on COPD due to its significant prevalence in the country, affecting nearly 100 million individuals (Wang et al., 2018). This focus allows our results to mirror the broader challenges faced in managing chronic diseases within a large population. Our findings offered insights into potential strategies to enhance patient attendance at follow-up care in clinical settings, thus improving disease control.

## 2 Materials and methods

### 2.1 Study design

In this retrospective multicenter cohort study, data were collected from the CWPC platform. The study initiated by the China-Japan Friendship Hospital and extending to 1,223 hospitals across 29 provinces (Cough and Wheeze Pharmaceutical Care Clinic, 2022). Pharmacists from the CWPC across all participating hospitals adhere to a standard operating procedures manual and undergo regular training. After visiting a respiratory clinician, patients would go to CWPC and receive comprehensive medication management services, including drug education, polypharmacy management, training in inhaler techniques, and health education from pharmacists, which are offered at no charge. Patients were encouraged to maintain ongoing engagement with CWPC for continuous support in medication management and to enhance their inhaler techniques.

Data collection for this study spanned from January 2021 to November 2022. This study was conducted in accordance with the Declaration of Helsinki and was approved by the Clinical Research Ethics Committee at the China-Japan Friendship Hospital (2021-84-K49-1). For data collection, patients’ information was de-identified. The study documented participants’ demographic and clinical characteristics, visit dates, health conditions, prescribed medications, and healthcare service utilization upon entry. The eligibility criteria were: 1) aged ≥18 years; 2) previously diagnosed with COPD. Additionally, inpatients, patients without medication regimen records and visit dates were excluded (Supplementary Figure S1).

### 2.2 Attendance of follow-up care

Similar to previous studies (Kim et al., 2024; Wong et al., 2024), the study defined follow-up care attendance as an outpatient visit at CWPC subsequent to the initial baseline attendance. The length of follow-up time was measured by counting the number of months between the initial visit and the subsequent visit at CWPC.

### 2.3 Medication regimen complexity

We utilized the Medication Regimen Complexity Index (MRCI) to assess the complexity of COPD medication regimens, aggregating scores for dosage form, frequency, and additional instructions (George et al., 2004). Higher MRCI scores reflect greater complexity in medication management. Our study focused on evaluating the MRCI for medication regimens prescribed by respiratory physicians.

For calculating the dose form score, 0 was assigned if no COPD-specific medication was prescribed. In cases where the medication record mentioned only the method of intake without specifying the medication name (e.g., “other oral drug”), the lowest possible score was assigned (e.g., one for a tablet or capsule). The dose frequency score was treated as missing data if the record did not specify a regular intake frequency (e.g., “take the medication immediately”). Likewise, if no special instructions were indicated for a medication, a score of 0 was assigned for the additional instruction component. A trained pharmacist calculated the MRCI scores, which were subsequently verified by one of the study authors to ensure accuracy. Following this verification, the scores were finalized and utilized as the definitive MRCI scores for our analysis.

Without established MRCI benchmarks and considering that a high-complexity regimen often entails polypharmacy (defined as the use of five or more medications) (Varghese et al., 2024), we identified a threshold of 15.0 points indicative of high regimen complexity. The total MRCI score larger than 15.0 was defined as high MRCI score group. This threshold was based on including a triple combination inhaler (contributing 7.0 points from its dosage form, frequency, and indication) and four additional medications, each adding a minimum of 2.0 points for their dosage form and frequency. Moreover, a dosage form score larger than 10.0 was defined as a high score, suggesting potential inhaler device polypharmacy (Metered dose inhalers [MDI] and two other inhalants) (Negewo et al., 2017). Scores larger than 5.0 and 1.0 were high in dose frequency and instruction score, respectively.

### 2.4 Sample size calculation

Given the novel nature of our study and the lack of similar research in the literature, we defined the attendance rates for both groups based on pharmacists’ experience at CWPC. Our primary outcome focused on follow-up care attendance rate. We hypothesized that the follow-up care attendance rate would be 0.11 in the group with high medication complexity, and 0.14 in the group with low medication complexity. Drawing from clinical insights, we further determined the proportion of patients in the high versus low medication complexity groups to be 1:3. Aiming for a power of 0.8 and a double-sided significance level of 0.05, and accounting for a 20% dropout rate, we determined that a sample size of 1,464 for the high medication complexity group and 4,878 for the low medication complexity group was required. In total, the study required 6,342 patients.

### 2.5 Covariates and subgroup indicators

The study includes the following baseline covariates and subgroup indicators: 1) patient demographic characteristics (age, sex, ethnicity), 2) clinical characteristics (body mass index, systolic blood pressure, heart rate, COPD Assessment Test), 3) health behavior and history of health conditions (smoking, comorbidity), 4) healthcare use (hospital level, hospital region, and insurance status). COPD comorbidity was defined as the presence of one or more health conditions besides COPD. The study further identified 17 health conditions considered as comorbidities, ranging from physical comorbidities like hypertension and asthma to mental comorbidities such as anxiety and depression. These previously diagnosed comorbidities were identified through either self-reports from the patients or extracted from their medical records. A detailed breakdown of these comorbidities is presented in Supplementary Table S1.

### 2.6 Statistical analysis

Descriptive statistics and distributional graphs were used to describe the study population and the MRCI scores within each group. Bivariate comparisons of patient and hospital characteristics between high and low levels of MRCI scores were evaluated using the Mann-Whitney U test or Chi-Square test. Due to covariates missing data ranging from 10.8% to 24.6%, we applied multiple imputation by chained equations, generating 10 imputed datasets for the analysis.

The report included monthly intervals between visits and from the initial visit to the final observation date. We used Cox proportional hazard regression to calculate the adjusted hazard ratios (HRs) and their corresponding 95% confidence intervals (CIs) with medication complexity. We first tested the association of total MRCI score with follow-up care attendance. The low medication complexity group (MRCI score ≤15.0) was the reference category. Subsequently, we examined the association between MRCI subscores and follow-up care attendance. For the MRCI subscores, low dose form (score ≤10.0), low dose frequency (score ≤5.0), and low additional instruction (score ≤1.0) were the reference categories. Statistical models were adjusted for demographic characteristics (age and sex), clinical characteristics (body mass index, systolic blood pressure, heart rate, and COPD Assessment Test), comorbidity status, smoking status, and healthcare use (hospital level, hospital location, insurance coverage status). Analysis stratification was based on patient and hospital characteristics. For sensitivity analysis, missing monthly intervals between two consecutive visits or the baseline visit up to the last recorded date were imputed with the median monthly interval observed in the hospital. Analyses were conducted using Stata statistical software version 17.0 (StataCorp).

## 3 Results

### 3.1 Participant characteristics

Our study analyzed data from 16,684 patients, of which 2,306 (13.8%) returned for medication management. The attendance rate for the high medication complexity group was 11.9% compared to 14.3% for the low medication complexity group. For those who attended follow-up care, the median number of months between two visits was 1.3 (IQR, 1.0–3.0). Among all the patients, 3,385 (20.3%) had high MRCI scores (MRCI scores >15.0). The median age for the study population was 71.0 (interquartile range [IQR], 65.0–78.0) years. 4,119 (24.7%) patients were female, 13,340 (80.0%) patients visited tertiary hospitals, and 8,971 (53.8%) patients were with COPD comorbidities.

### 3.2 MRCI scores and subscores

**FIGURE 1:**
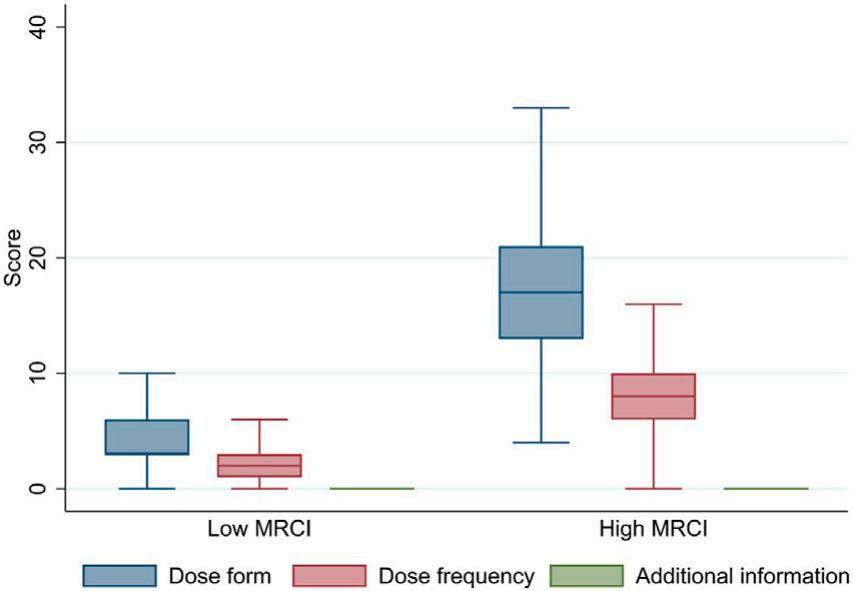
Characteristics of Subscores by MRCI Level^a^. ^a^In the box plots, lines within boxes denote medians, ends of boxes denote interquartile ranges, and error bars denote the lower and upper adjacent values. High MRCI group was with score >15.0. Low MRCI group was with score ≤15.0. Abbreviations: MRCI, Medication Regimen Complexity Index.

The median (IQR) MRCI scores for patients were 8.0 (5.0–14.0) overall, 5.0 (5.0–9.0) for the low MRCI group, and 24.0 (19.0–31.0) for the high MRCI group. Table 1 showed that compared to the low MRCI group, the high MRCI group had a larger proportion of older patients, females, patients with COPD, and patients who visited non-tertiary hospitals. The median (IQR) MRCI subscores were 5.0 (3.0–9.0) for dose form, 2.0 (2.0–5.0) for dose frequency, and 0.0 (IQR: 0.0 to 0.0) for additional instruction. Figure 1 illustrates the distributions of subscores by MRCI score level. The dose form subscore contributed most to medication complexity, particularly at the high MRCI level. For the prescribed dose forms, inhalants took up 42.3%, followed by nebulizers at 19.3%.

**TABLE 1:** Baseline characteristics of patients with COPD by MRCI score.

Patient and hospital characteristics	Total, no. (%) (N = 16,684)	High MRCI score (MRCI >15.0), no. (%) (N = 3,385)	Low MRCI score (MRCI ≤15.0), no. (%) (N = 13,299)	*p*-value^a^
Age (years), median (IQR)	71.0 (65.0–78.0)	72.0 (66.0–79.0)	71.0 (64.0–78.0)	<0.001
Sex of birth				0.020
Female	3,729 (22.3)	807 (23.8)	2,922 (22.0)	
Male	12,955 (77.7)	2,578 (76.2)	10,377 (78.0)	
Ethnicity				<0.001
Han	16,224 (97.2)	3,236 (95.6)	12,988 (97.7)	
Others	460 (2.8)	149 (4.4)	311 (2.3)	
BMI (kg/m^2^), median (IQR)	22.0 (19.8–24.2)	22.0 (19.6–24.2)	22.0 (19.9–24.2)	0.093
SBP (mmHg), median (IQR)	130 (120–141)	130 (119–142)	130 (120–141)	0.556
HR (times/min), median (IQR)	86 (78–97)	89 (80–101)	85 (78–96)	<0.001
CAT, median (IQR)	22 (16–26)	24 (20–28)	21 (16–26)	<0.001
COPD comorbidity				<0.001
With	8,971 (53.8)	2,137 (63.1)	6,834 (51.4)	
Without	7,713 (46.2)	1,248 (36.9)	6,465 (48.6)	
Hospital level				<0.001
Tertiary hospital	13,340 (80.0)	2,580 (76.2)	10,760 (81.9)	
Non-tertiary hospital	3,344 (20.0)	805 (23.8)	2,539 (19.1)	
Hospital region				0.001
Southern	12,208 (73.2)	2,400 (70.9)	9,808 (73.8)	
Northern	4,476 (26.8)	985 (29.1)	3,491 (26.3)	
Insurance status				<0.001
Insurance covered	15,065 (90.3)	3,227 (95.3)	11,838 (89.0)	
Insurance uncovered	1,619 (9.7)	158 (4.7)	1,461 (11.0)	
Smoking status				0.548
Current or previous	9,308 (55.8)	1,904 (56.3)	7,404 (55.7)	
Nonsmoker	7,376 (44.2)	1,481 (43.7)	5,895 (44.3)	
Attended follow-up care				
Yes	2,306 (13.8)	402 (11.9)	1,904 (14.3)	0.015
No	14,378 (86.2)	2,983 (88.1)	11,395 (85.7)	

Abbreviations: MRCI, medication regimen complexity index; BMI, body mass index; SBP, systolic blood pressure; HR, heart rate; COPD, chronic obstructive pulmonary disease; CAT, COPD, assessment test.

^a^ Calculated with the Mann-Whitney U test or Chi-Square test.

### 3.3 Association of medication complexity and follow-up care attendance

The median follow-up time was 11.4 months. Compared with patients who had low medication complexity, a higher MRCI score (>15.0) was associated with a decreased likelihood of attending follow-up care, with HRs of 0.82 (95% CI, 0.74–0.91). When looked into MRCI subscores, higher dose form complexity (score >10.0) was associated with a decreased likelihood of attending follow-up care, with HRs of 0.49 (95% CI, 0.43–0.57). Conversely, higher dose frequency (score >5.0) and instruction complexity (score >1.0) were associated with an increased likelihood of attending follow-up care, with HRs of 1.56 (95% CI, 1.37–1.77) and 1.61 (95% CI, 1.30–1.99), respectively (Table 2).

**TABLE 2:** Associations between medication regimen complexity and follow-up care attendance.

High vs. low score (N = 16,684)	Unadjusted		Demographic characteristics adjusted^a^		Demographic and clinical characteristics adjusted^b^		Fully adjusted^c^	
	Hazard ratio (95% CI)	*p*-Value	Hazard ratio (95% CI)	*p*-Value	Hazard ratio (95% CI)	*p*-Value	Hazard ratio (95% CI)	*p*-Value
MRCI score (High score >15.0)	0.82 (0.74–0.91)	<0.001	0.82 (0.74–0.92)	<0.001	0.82 (0.73–0.91)	<0.001	0.87 (0.78–0.97)	0.010
Dose form score (High score >10.0)	0.49 (0.43–0.57)	<0.001	0.49 (0.43–0.57)	<0.001	0.49 (0.42–0.56)	<0.001	0.52 (0.45–0.60)	<0.001
Dose frequency score (High score >5.0)	1.56 (1.37–1.77)	<0.001	1.56 (1.37–1.77)	<0.001	1.55 (1.37–1.76)	<0.001	1.54 (1.36–1.75)	<0.001
Indication score (High score >1.0)	1.61 (1.30–1.99)	<0.001	1.61 (1.30–1.99)	<0.001	1.60 (1.29–1.99)	<0.001	1.44 (1.16–1.79)	0.001

^a^ Adjusted for age and sex.

^b^ Adjusted for age, sex, body mass index, systolic blood pressure, heart rate, and COPD, assessment test.

^c^ Adjusted for age, sex, ethnicity, body mass index, systolic blood pressure, heart rate, COPD, assessment test; COPD, comorbidity status, hospital level, hospital location, insurance coverage status, and smoking status.

After adjusted for potential confounders (demographic and clinical characteristics, comorbidity status, smoking status, and healthcare use), HRs remained significant. Those prescribed medications with higher complexity regimens were 13% less likely to return to medication management (HR, 0.87; 95% CI, 0.78–0.97) compared to patients with lower complexity regimens. Analysis of the subscores revealed that a higher dose form score significantly decreased the likelihood of attending follow-up care (HR, 0.52; 95% CI, 0.45–0.60), whereas higher scores in dose frequency (HR, 1.54; 95% CI, 1.36–1.75) and additional instructions (HR, 1.44; 95% CI, 1.16–1.79) were associated with an increased likelihood of attending follow-up sessions (Table 2). These main analysis results were corroborated by the sensitivity analysis (Supplementary Table S2), demonstrating consistent findings.

### 3.4 Medication complexity and follow-up care attendance by patient’s and hospital’s characteristics

**FIGURE 2:**
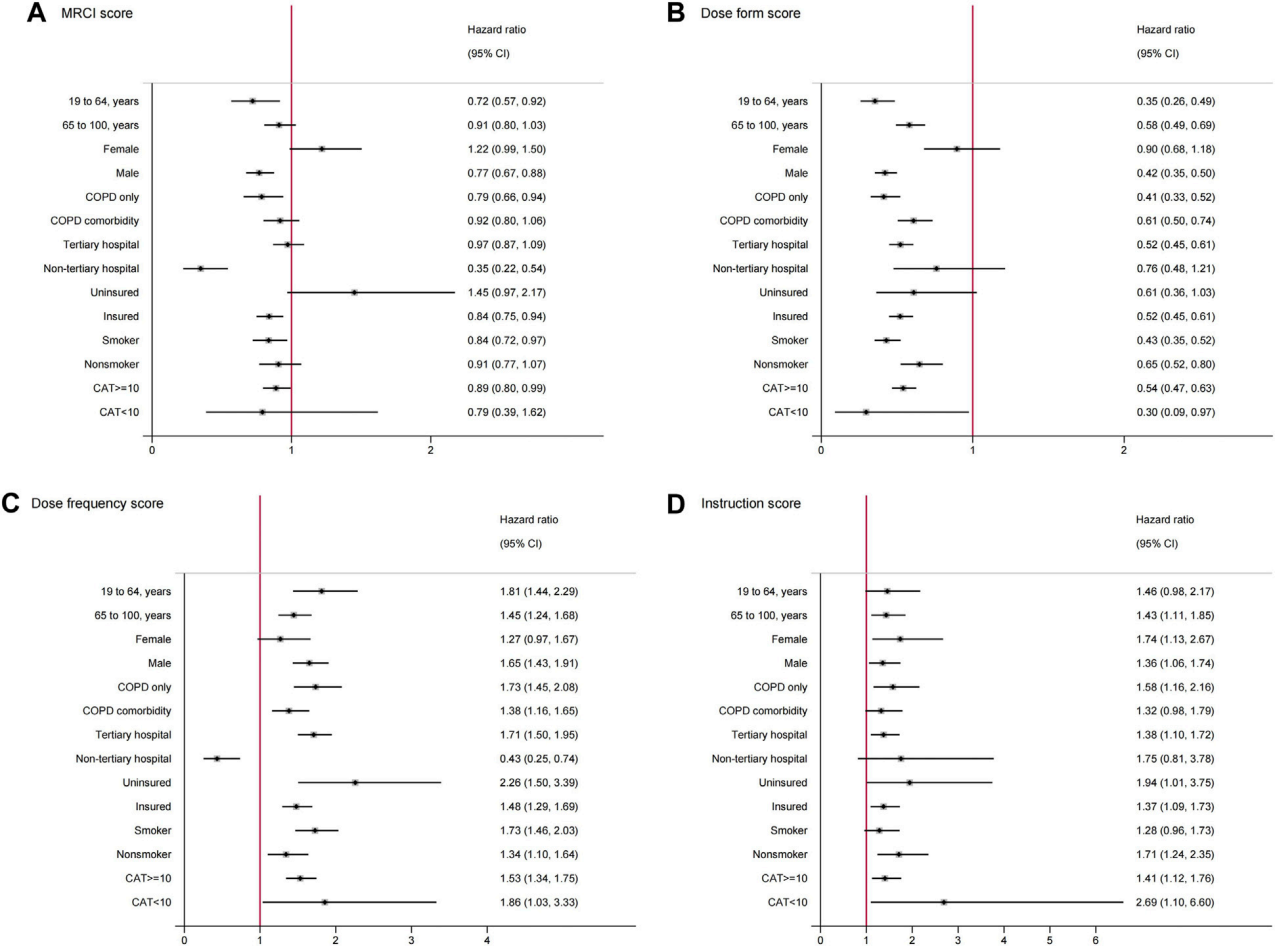
Subgroup analysis for the associations between medication regimen complexity and follow-up care attendance^a^. ^a^Graphs showed the hazard ratio and 95%CI for **(A)**, high vs. low MRCI score; **(B)**, high vs. low dose form score; **(C)**, high vs. low dose frequency score; **(D)**, high vs. low instruction score. Dots represent the hazard ratios, error bars represent the 95%CIs. Adjusted for age, sex, ethnicity, body mass index, systolic blood pressure, heart rate, COPD Assessment Test, COPD comorbidity status, hospital level, hospital location, insurance coverage status, and smoking status. Abbreviations: MRCI, Medication Regimen Complexity Index; COPD, Chronic Obstructive Pulmonary Disease; CAT, COPD Assessment Test.

We further investigated the association between medication complexity and follow-up care attendance by patients and hospital’s characteristics. After adjusted for all the covariates, we observed that among patients younger than 65 years (HR, 0.72; 95% CI, 0.57–0.92), male patients (HR, 0.77; 95% CI, 0.67–0.88), and patients who visited non-tertiary hospitals (HR, 0.35; 95% CI, 0.22–0.54), higher medication complexity was strongly associated with less likelihood of follow-ups (Figure 2A). Upon analyzing in stratified groups with subscores, we also observed that among patients younger than 65 years (HR, 0.35; 95% CI, 0.26–0.49) and male patients (HR, 0.42; 95% CI, 0.35–0.50), the likelihood of attending follow-up care decreased when patients used complex dose forms (Figure 2B). For patients who visited non-tertiary hospitals, an increased frequency of doses was associated with a reduced likelihood of attending follow-up care (HR, 0.43; 95%CI, 0.25 to 0.74, Figure 2C).

The results of the sensitivity analysis were similar to those of the main analysis (Supplementary Figure S2).

## 4 Discussion

In this population-based retrospective cohort study, we found that individuals prescribed complex medication regimens (MRCI score >15.0), particularly those with intricate dosing forms (score >10.0), demonstrated lower attendance to follow-up care. In contrast, patients with medication regimens characterized by higher dose frequencies (score >5.0) or additional instructions (score >1.0) were more likely to attend follow-up care. Notably, our analysis identified that 20.3% of the study participants had high-complexity medication regimens (MRCI score >15.0), with the complexity of COPD-specific medication primarily attributed to the complexity in dose forms.

To the best of our knowledge, the study is the first to formally evaluate the associations between medication complexity and follow-up care attendance in pharmaceutical clinic settings across all levels of hospitals. Specifically, the CWPC is the first and largest pharmaceutical clinic dedicated to managing respiratory medications in China. This unique position enabled us to conduct an in-depth analysis of patient attendance patterns at these outpatient care facilities.

In order to promote care continuity, previous research explored external measures like reminders (Sawyer et al., 2002) or third-party involvement (Tait et al., 2004). Our study shed light on an alternate avenue for enhancing follow-up care: simplifying medication regimens. Earlier research showed that the complexity of COPD medication regimens was mainly due to their dosage formulations (Negewo et al., 2017). Specifically, inhaler device polypharmacy is highly prevalent (Negewo et al., 2017). In our study, 21.7% (3,626 out of 16,684) patients utilizing two or more types of inhalers. This underscores the necessity for dose form simplification, a recommendation echoed by the latest GOLD guidelines, which favor single inhaler therapy for its ease of use and potential efficacy advantages over the use of multiple inhalers (Venkatesan, 2024). Notably, our findings suggested a gap in the current practice at CWPC, where the focus was solely on medication management without addressing patient concerns about regimen complexity to physicians. Our findings highlighted the importance of the collaboration between pharmacists and respiratory physicians to provide the best medication care to patients in terms of effectiveness, safety, and simplicity.

Our study uncovered a notably low rate of follow-up visit attendance, particularly among patients prescribed complex medication regimens. This finding underscores the urgent need to bolster awareness of follow-up care within the framework of chronic disease management. The diminished propensity for follow-up care among patients with high medication complexity may not come as a surprise to many practitioners. Previous research revealed that medication adherence and correct usage among patients with COPD were notably low (Chrystyn et al., 2017; Wu et al., 2024), which often attributed to the complexity of medication regimes, including the use of inhalers and the prescription of multiple drugs (Albayrak et al., 2023). As patients struggle with intricate treatment plans, it could lead to ineffective disease control. Rather than seeking advice on proper medication usage, patients facing uncontrolled symptoms may seek to change their treatment plan altogether (Krigsman et al., 2007; López-Pintor et al., 2021). Such behavior could lead to a lower rate of follow-up visits. While switching medication regimens and non-adherence can relate to higher costs and a waste of medication resources (Kane et al., 2008; Lloyd et al., 2019). Consequently, it becomes critical to ensure that patients are consistently reminded of the importance of long-term medication monitoring and management guided by healthcare professionals. An intriguing facet of our research revealed that patients prescribed medications with high dose frequencies or additional instructions were, in fact, more inclined to attend follow-up care. This suggests that the frequent and meticulous nature of their treatment regimen might serve as a continual reminder of the importance of medication management (Stewart et al., 2023), thus motivating them to seek professional advice and support.

Evidence indicated that comprehensive medication management positively impacts the management of COPD, particularly in enhancing patients’ inhaler technique education and medication adherence (Hesso et al., 2016). Studies underscore the necessity of providing patients with repeated instructions on correct inhaler use, rather than treating this guidance as a one-off intervention, to ensure sustainable health outcomes (Hesso et al., 2016). Despite the aim of our study to offer ongoing pharmaceutical care at the CWPC, there was a notable low attendance rate for follow-up care. The trend echoed in medication management programs globally. For instance, in England, a pharmacist-led comprehensive medication care program saw a 35% dropout rate within 6 months (Wright et al., 2015), while in Sweden, the likelihood of attending a regular follow-up visit stood at just 39.1% (Sandelowsky et al., 2022). Patients lacking regular COPD follow-up visits reported significantly higher use of oral corticosteroids and respiratory antibiotics, alongside reduced maintenance treatment (Sandelowsky et al., 2022). Similarly, Randomized Controlled Trials (RCTs) focusing on COPD care reported dropout rates ranging between 14.6% and 25.6%, with longer-duration programs experiencing higher dropout rates (Khdour et al., 2009; Coultas et al., 2016; Kessler et al., 2018; North et al., 2020). Similar to our study, these trials applied uniform interventions across all complexity levels of the medication regimen. However, our data indicated that patient engagement in follow-up care varied according to the complexity of their medication regimens. Accordingly, future strategies should prioritize intensified education for those with high medication complexity. Moreover, fostering direct communication to clarify treatment plans might improve adherence to follow-up care (Osterberg et al., 2005). Such a targeted approach promises to better support patients with COPD, maximizing the benefits of educational programs and effectively managing the condition.

Our study revealed that among male patients, individuals younger than 65 years, and those without additional comorbid conditions, the impact of medication complexity on follow-up care attendance was more pronounced. Raising the attention on enhanced education for these group of patients. Intriguingly, in tertiary hospitals, the complexity of medication did not significantly impact patients’ follow-up attendance. While in non-tertiary hospitals, a higher medication complexity was associated with a decreased likelihood of follow-up. Given that CWPC clinics provided standardized pharmaceutical care at no cost, the variance in follow-up patterns could be attributed to differing healthcare needs among patients (Liu et al., 2020). Specifically, those with more complex medication regimens might experience uncontrolled symptoms (Federman et al., 2021), prompting them to seek care from higher-level hospitals rather than returning to the original healthcare provider. Strengthening primary healthcare systems to more effectively meet the needs of patients with chronic diseases might improve care continuity.

### 4.1 Limitations of the study

Our study faces several limitations. Firstly, the quality of the data may have introduced inaccuracies due to incomplete clinical records. Although our sensitivity analysis did not reveal any significant difference between complete and incomplete follow-up records, there could be potential bias. Secondly, this research focused on assessing the influence of medication complexity on follow-up attendance. Moving forward, we plan to conduct a cluster randomized controlled trial (RCT) to investigate the effects of comprehensive, regular pharmaceutical care on the health outcomes of patients with COPD. This future work aims to build on our current findings and further elucidate the role of continuous pharmaceutical care in managing COPD effectively.

### 4.2 Conclusion

Patients with high medication complexity, especially with complex dose forms, was less likely to attend follow-up care. It is imperative that future efforts in chronic disease management prioritize the simplification of medication regimens. Moreover, there is a pressing need to enhance education on the significance of follow-up care in medication management, especially for patients with complex medication regimens.

## Data Availability

Data were collected from the platform of Cough and Wheeze Pharmaceutical Care Clinic (CWPC) (https://hsyuntai.cn/cwpc). Data are publicly available upon request.
