# Supplementary material for: Association between medication complexity and follow-up care attendance: insights from a retrospective multicenter cohort study across 1,223 Chinese hospitals
Source: Front Pharmacol. 2024 Jul 29;15:1448986. doi: 10.3389/fphar.2024.1448986 (PMC11317271; doi:10.3389/fphar.2024.1448986)
Supplement: Supplementary file 1 [file DataSheet1.docx]

Supplementary Material

# Supplementary Figures and Tables

## Supplementary Figures


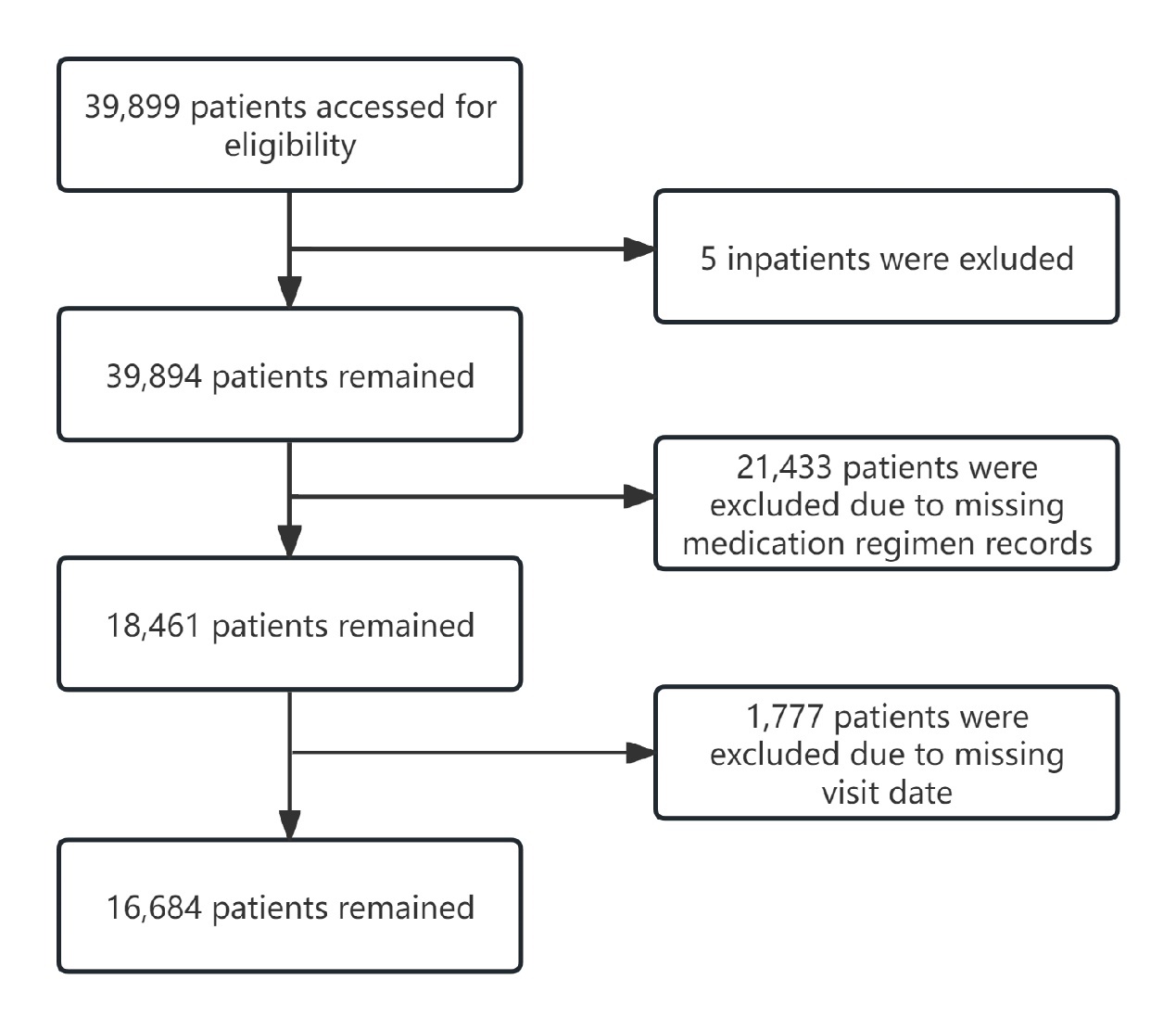


**Supplementary Figure 1** **Flowchart of study population inclusion and exclusion**


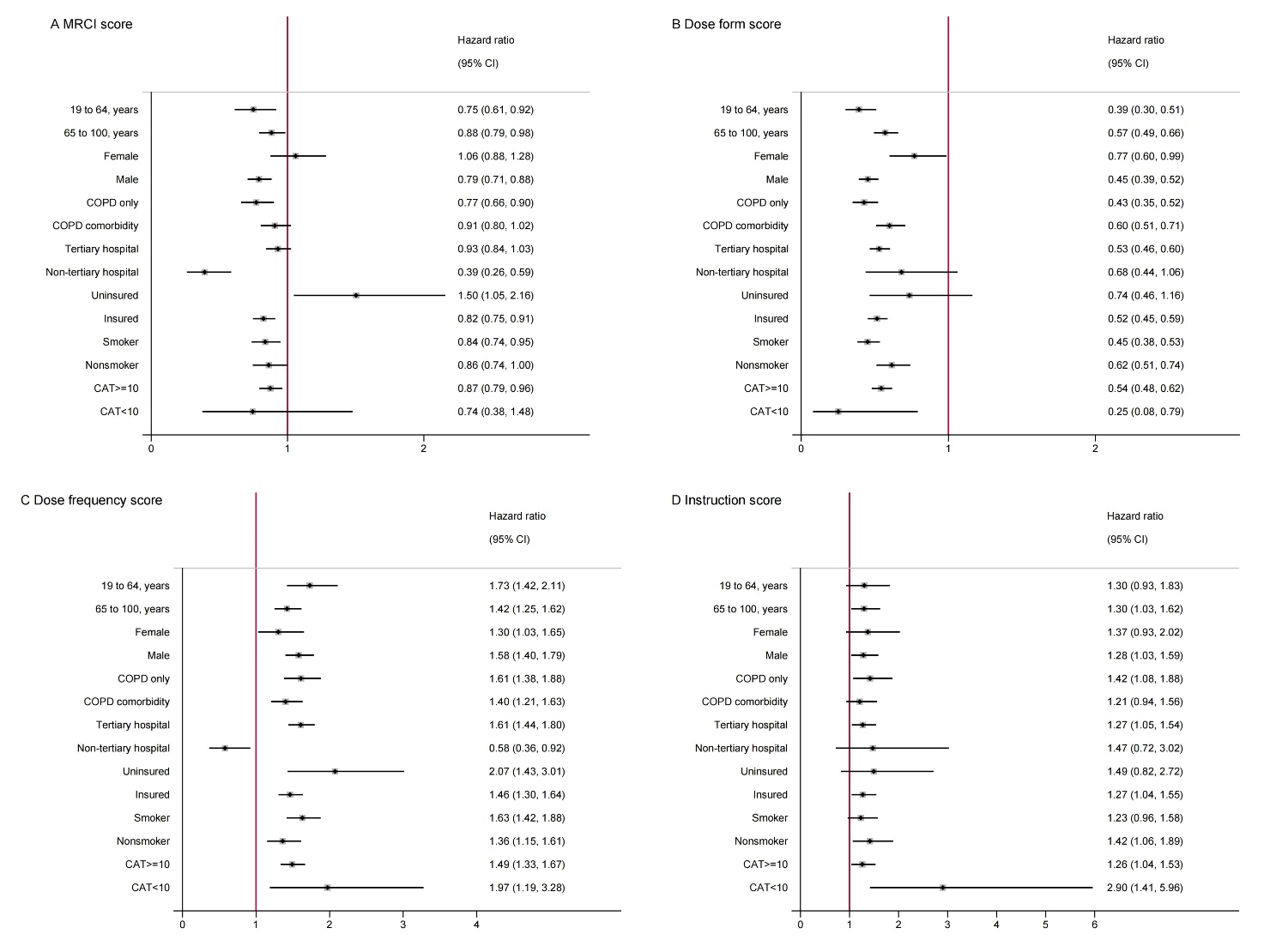


**Supplementary Figure 2** **Subgroup analysis for the associations between medication regimen complexity and follow-up care attendance with imputed follow-up period^a^**

^a^Graphs showed the hazard ratio and 95%CI for A, high vs low MRCI score; B, high vs low dose form score; C, high vs low dose frequency score; D, high vs low instruction score. Dots represent the hazard ratios, error bars represent the 95%CIs. Adjusted for age, sex, ethnicity, body mass index, systolic blood pressure, heart rate, COPD Assessment Test, COPD comorbidity status, hospital level, hospital location, insurance coverage status, and smoking status.

Abbreviations: MRCI, Medication Regimen Complexity Index; COPD, Chronic Obstructive Pulmonary Disease; CAT, COPD Assessment Test.

## Supplementary Tables

**Supplementary Table 1 The 17 Health conditions included in the study**

| No. | Disease name |
| --- | --- |
| 1 | Anxiety |
| 2 | Depression |
| 3 | Lung Cancer |
| 4 | Hypertension |
| 5 | Diabetes |
| 6 | Coronary Heart Disease |
| 7 | Hyponatremia |
| 8 | Hypoproteinemia |
| 9 | Hypothyroidism |
| 10 | Pulmonary Heart Disease |
| 11 | Electrolyte Imbalance |
| 12 | Gastritis |
| 13 | Hypokalemia |
| 14 | Hyperuricemia |
| 15 | Asthma |
| 16 | Hyperthyroidism |
| 17 | Gastroesophageal Reflux Disease |

**Supplementary Table 2 Associations between medication regimen complexity and follow-up care attendance with imputed follow-up period**

| High vs low score (N=18,448) | Unadjusted | |  | Demographic characteristics adjusted^a^ | |  | Demographic and clinical characteristics adjusted^b^ | |  | Fully adjusted^c^ | |
| --- | --- | --- | --- | --- | --- | --- | --- | --- | --- | --- | --- |
|  | Hazard ratio (95% CI) | P value |  | Hazard ratio (95% CI) | P value |  | Hazard ratio (95% CI) | P value |  | Hazard ratio (95% CI) | P value |
| MRCI score (High score > 15.0) | 0.80 (0.73 to 0.88) | <0.001 |  | 0.80 (0.73 to 0.88) | <0.001 |  | 0.80 (0.72 to 0.88) | <0.001 |  | 0.85 (0.77 to 0.94) | 0.001 |
| Dose form score (High score > 10.0) | 0.49 (0.43 to 0.55) | <0.001 |  | 0.49 (0.43 to 0.55) | <0.001 |  | 0.48 (0.43 to 0.55) | <0.001 |  | 0.52 (0.46 to 0.59) | <0.001 |
| Dose frequency score (High score > 5.0) | 1.52 (1.37 to 1.70) | <0.001 |  | 1.52 (1.37 to 1.70) | <0.001 |  | 1.52 (1.36 to 1.69) | <0.001 |  | 1.51 (1.35 to 1.68) | <0.001 |
| Indication score (High score > 1.0) | 1.45 (1.20 to 1.74) | <0.001 |  | 1.44 (1.20 to 1.74) | <0.001 |  | 1.44 (1.20 to 1.73) | <0.001 |  | 1.31 (1.08 to 1.58) | 0.005 |

^a^Adjusted for age and sex.

^b^Adjusted for age, sex, body mass index, systolic blood pressure, heart rate, and COPD Assessment Test.

^c^Adjusted for age, sex, ethnicity, body mass index, systolic blood pressure, heart rate, COPD Assessment Test, COPD comorbidity status, hospital level, hospital location, insurance coverage status, and smoking status.
